# 超高效液相色谱-串联质谱法测定运动营养食品中60种类固醇激素

**DOI:** 10.3724/SP.J.1123.2024.03020

**Published:** 2024-12-08

**Authors:** Xiaoming CAI, Shaoming WU, Qing YU, Hehe HUANG, Qiuping ZHENG, Menghang HE, Ming DAI, Liqun OUYANG

**Affiliations:** 福建省产品质量检验研究院食品检验研究所,国家加工食品质量检验检测中心,福建 福州 350002; Institute of Food Inspection, Fujian Inspection and Research Institute for Product Quality, National Quality Supervision and Testing Center for Processed Food, Fuzhou 350002, China

**Keywords:** 超高效液相色谱-串联质谱, QuEChERS, 类固醇激素, 运动营养食品, ultra performance liquid chromatography-tandem mass spectrometry (UPLC-MS/MS), QuEChERS, steroid hormones, sports nutrition food

## Abstract

基于QuEChERS,结合超高效液相色谱-串联质谱(UPLC-MS/MS)技术,建立了一种同时测定运动营养食品中60种类固醇激素的分析方法。样品以1.0%(v/v)甲酸乙腈提取,氯化钠进行分层,150 mg PSA和150 mg C_18_粉末净化,净化液经氮气吹至近干后用50%甲醇水溶液复溶,在Waters Acquity UPLC HSS T_3_分析柱上,以0.1%(v/v)甲酸水溶液(含10 mmol/L乙酸铵)-甲醇为流动相,电喷雾离子源(ESI)正离子模式下进行UPLC-MS/MS多反应监测(MRM)测定。在最优的仪器参数及前处理条件下进行方法验证,60种类固醇激素在1.0~100 ng/mL范围内具有良好的线性关系,相关系数(*r*)均大于0.99;方法的检出限(LOD)和定量限(LOQ)分别为0.2~0.8 μg/kg和0.6~2.4 μg/kg。以乳清蛋白粉(固体)、果胶蛋白肽(半固体)、运动营养液(液体)为空白基质,进行3个水平的加标回收试验(*n*=6), 60种类固醇激素的平均回收率为73.7%~112.7%,相对标准偏差(RSD)为3.2%~10.1%。将新开发的方法应用于13批次实际样品的测定,在其中1份样品中检出禁用药物(勃地酮),含量为14.6 μg/kg,说明运动营养食品中确有非法添加类固醇激素的现象,后续应加强对运动营养食品中该类固醇类激素的监控。该方法简单高效,灵敏度高,重复性好,适用于运动营养食品中类固醇激素的快速筛查和定量分析,为运动营养食品中类固醇激素的日常监督提供技术支撑。

## Abstract (en)

Recent societal developments and improving living standards have witnessed a growing enthusiasm for sport and fitness along with a boom in sports nutrition foods. However, rapid industrial development has led to product quality and safety issues becoming increasingly important. Steroid hormones exhibit physiological effects that include promoting protein synthesis, increasing muscle mass, and reducing body fat. Driven by vested interests, some unscrupulous traders illegally add steroid hormones to sports nutrition foods to improve sports performance and relieve muscle pain, which poses a potential threat to human health. Therefore, establishing a method for determining steroid hormones in sports nutrition foods is of great practical importance.

In this study, an analytical method for the simultaneous determination of 60 steroid hormones in sports nutrition foods was developed using QuEChERS combined with ultra performance liquid chromatography-tandem mass spectrometry (UPLC-MS/MS). The MS parameters and chromatographic conditions were optimized for the 60 steroid hormones, and the extraction solvents and purification methods were systematically optimized to facilitate rapid screening and accurate quantitative analysis of these hormones in sports nutrition foods. Samples were extracted with 1.0% formic acid in methanol, partitioned with sodium chloride, and purified using 150 mg of PSA and 150 mg of C_18_ powder. The clean-up solution was evaporated to near dryness under a stream of nitrogen and the residue was reconstituted with 50% methanol aqueous solution. The 60 target compounds were separated on a Waters Acquity UPLC HSS T_3_ column (100 mm×2.1 mm, 1.8 μm) with 0.1% (v/v) formic acid aqueous solution (containing 10 mmol/L ammonium acetate) and methanol as the mobile phases. The analytes were determined by multiple reaction monitoring (MRM) in positive electrospray ionization mode (ESI^+^) and quantified using the external standard method. The method was validated under the optimized conditions, with the 60 steroid hormones found to exhibit good linearities in the 1.0-100 ng/mL range with correlation coefficients (*r*) greater than 0.99. The limits of detection (LODs) and limits of quantification (LOQs) were 0.2-0.8 μg/kg and 0.6-2.4 μg/kg, respectively. At three spiked levels on whey protein powder (solid), pectin protein peptide (semi-solid) and energy drink (liquid) as blank matrix, the mean recoveries of the 60 steroid hormones were 73.7%-112.7% with relative standard deviations (RSDs) of 3.2%-10.1% (*n*=6). Thirteen batches of commercially available sports nutrition products were screened using the newly developed method, with five positive samples detected. Progesterone (an endogenous hormone) was detected at levels of 12.6-25.4 μg/kg in four samples of whey protein powder, and boldenone (which is a prohibited drug for athletes) was detected at 14.6 μg/kg in one sports nutrition liquid, confirming that illegal steroid hormones are indeed added to sports nutrition foods; consequently, relevant national departments need to strengthen their monitoring of such substances in such foods. The developed method is simple, efficient, sensitive, reproducible, and suitable for the rapid screening and quantitative analysis of steroid hormones in sports nutrition foods, thereby providing technical support for the daily supervision of steroid hormones in these foods.

近年来,随着社会的发展和生活水平的提高,人们对运动健身的热情日益高涨,这促进了运动营养食品市场的蓬勃发展^[1]^,但随之而来的产品质量安全问题也日益凸显,尤其是国外研究者Plotan等^[2]^在多款运动营养食品中检测出类固醇激素化合物,这引起了人们的广泛关注。类固醇激素包括糖皮质激素和性激素两大类。其中性激素可以促进肌肉生长和力量增强,提高运动表现,促进康复和恢复以及影响心理状态,对运动员的训练和比赛成绩产生积极影响^[3⇓-5]^;而糖皮质激素则具有强大的抗炎作用,可减轻炎症反应,缓解肌肉疼痛和不适,促进康复^[6]^。因此在利益的驱动下,一些不法商家将类固醇激素非法添加至运动营养食品中以提高产品的性能。然而,过量摄入类固醇激素会造成人体内分泌失调、肾脏损伤,甚至患癌等风险^[7⇓⇓⇓-11]^。因此,欧盟、美国、日本明确禁止在动物源性食品中使用类固醇激素药物^[12]^,我国农业部公告250号《动物性食品中兽药最高残留限量》也规定了动物源性食品中类固醇激素的最大残留限量^[13]^。而目前,对于运动营养食品中该类物质还未有相关的限量标准,缺乏有效的监督措施和配套的法律法规,更没有相应的国家标准检测方法。因此,建立一套高通量的运动营养食品中多组分类固醇激素的定性、定量检测方法具有十分重要的意义,对促进运动营养食品市场的健康发展也具有积极作用。

目前类固醇激素的分析主要针对的是动物源性食品,主要方法包括气相色谱法^[14]^、液相色谱法^[15]^、气相色谱-质谱法^[16,17]^和液相色谱-质谱法^[18⇓⇓⇓⇓-23]^,而针对运动营养食品的检测方法少有报道。气相色谱与液相色谱需将各组分完全分离才能进行准确定性、定量分析,对于多化合物的同时分析具有一定的局限性;气相色谱-质谱法虽然具有较高的灵敏度和抗干扰能力,但通常需要繁琐的衍生化过程。液相色谱-串联质谱同时具有高通量、高灵敏度、高选择性及高效性,已成为检测类固醇激素的主流方法。

类固醇激素的前处理方法主要包括固相萃取法^[24,25]^、固液萃取法^[26,27]^及QuEChERS法^[28⇓-30]^等,QuEChERS法由于操作简单、有机试剂用量少、效率高、成本低等特点,已成为高通量分析的主流前处理技术^[31]^。因此,本研究采用QuEChERS法,结合UPLC-MS/MS技术,建立了同时测定运动营养食品中60种类固醇激素的方法,并将所建立的方法应用于实际样品的测定,为相关监管部门提供技术支持和数据支撑。

## 1 实验部分

### 1.1 仪器与试剂

UPLC-MS/MS 8050超高效液相色谱-串联质谱仪(日本岛津公司); Avanti JXN-30/26智能型高效离心机(挪威Beckmann公司); KQ-200KDB超声波清洗机(昆山舒美有限公司); MS 3 basic旋涡混均器(德国IKA公司)。

60 种类固醇激素标准溶液购自天津阿尔塔公司,质量浓度均为1000 μg/mL,溶剂为甲醇,化合物名称见表1;甲酸、乙腈、甲醇(色谱纯,美国Merck公司);氯化钠、乙酸铵(分析纯,上海安谱公司), C_18_、PSA分散固相萃取剂(60 μm,苏州纳谱分析技术有限公司)。所用水均为屈臣氏蒸馏水。13批次运动营养食品购于福州各大超市,包括4份乳清蛋白粉(whey protein powder)、4份果胶蛋白肽(pectin protein peptide)、5份运动营养液(energy drink)。

**表1:** 60种类固醇激素的保留时间和质谱参数

No.	Analyte		*t*_R_/min			Parent ion (*m/z*)		Product ion (*m/z*)		Q1 Pre Bias/V		CE/ eV	Q3 Pre Bias/V
1	prednisolone (泼尼松龙)			4.701			361.2		147.2^*^		18	26	28
									343.4		16	13	17
2	hydrocortisone (氢化可的松)			4.717			363.3		327.2^*^		16	26	24
									105.2		16	52	11
3	methylprednisolone (甲泼尼松龙)			3.742			375.3		357.2^*^		10	35	17
									161.1		10	45	17
4	betamethasone (倍他米松)			5.205			393.2		355.2^*^		20	13	17
									372.9		18	11	26
5	dexamethasone (地塞米松)			5.205			393.2		237.1^*^		21	24	25
									147.1		11	32	29
6	triamcinolone (曲安西龙)			3.68			395.2		357.1^*^		12	15	17
									224.8		12	21	15
7	flumethasone (氟米松)			5.016			411.3		253.1^*^		18	18	27
									335.1		11	15	16
8	meprednisone (泼尼松)			4.293			359.3		147.1^*^		16	26	15
									341.2		15	12	16
9	cortisone (可的松)			4.419			361.3		163.1^*^		16	25	30
									121.2		12	32	24
10	beclomethasone (倍氯米松)			5.331			409.3		147.2^*^		18	29	16
									391.2		18	13	19
11	triamcinolone diacetate (曲安西龙双醋酸酯)			4.923			479.1		321.2^*^		22	17	24
									441.1		22	12	22
12	triamcinolone acetonide (曲安奈德)			8.042			435.7		339.1^*^		15	18	15
									397.7		30	12	14
13	prednisolone-21-acetate (泼尼松龙醋酸酯)			2.943			403.1		147.1^*^		28	26	27
									385.2		19	13	14
14	flurandrenolide (氟氢缩松)			5.676			437.1		121.1^*^		22	44	24
									181.1		21	54	18
15	fluorometholone (氟米龙)			5.521			377.0		279.1^*^		10	17	13
									321.3		18	16	23
16	deflazacort (地夫可特)			5.883			442.1		124.1^*^		21	47	12
									142.2		12	37	14
17	hydrocortisone acetate (氢化可的松醋酸酯)			5.458			405.1		309.1^*^		13	19	15
									327.2		18	18	16
18	fludrocortisone acetate (氟氢可的松醋酸酯)			5.427			423.3		239.2^*^		18	26	17
									121.2		11	37	12
19	prednisone 21-acetate (泼尼松醋酸酯)			5.38			403.1		146.9^*^		13	27	15
									295.1		13	17	20
20	cortisone acetate (可的松醋酸酯)			3.745			403.1		146.9^*^		11	25	13
									295.1		11	14	18
21	fluocinonide (氟轻松醋酸酯)			6.387			495.1		121.1^*^		23	35	12
									337.2		14	18	24
22	triamcinolone acetonide 21-acetate (曲安奈德醋酸酯)			6.418			477.1		321.2^*^		23	18	22
									339.2		23	17	26
23	hydrocortisone valerate (氢化可的松戊酸酯)			6.733			447.1		121.2^*^		21	30	25
									345.2		21	15	16
24	methylprednisolone acetate (甲基泼尼松龙醋酸酯)			6.026			417.1		253.1^*^		11	24	29
									399.2		19	11	28
25	eflone (氟米龙醋酸酯)			5.963			419.1		279.1^*^		15	18	13
									321.1		20	16	15
26	diflorasone diacetate (二氟拉松双醋酸酯)			6.215			495.1		279.1^*^		14	19	20
									317.1		14	16	22
27	budesonide (布地奈德)			6.513			431.1		147.1^*^		12	34	26
									413.2		21	14	20
28	betamethasone 21-acetate (醋酸倍他米松)			6.452			435.1		309.1^*^		21	17	22
									337.1		22	14	16
29	hydrocortisone-17-butyrate (氢化可的松丁酸)			6.199			433.2		121.1^*^		23	26	25
									345.2		13	13	24
30	dexamethasone-17-acetate (地塞米松醋酸酯)			6.973			435.1		309.1^*^		14	15	15
									337.1		14	15	16
31	mometasone furoate (莫米他松糠酸酯)			7.363			521.1		503.2^*^		26	13	24
									263.1		20	29	27
32	fluticasone propionate (氟替卡松丙酸酯)			6.782			501.0		293.2^*^		24	18	20
									313.2		26	14	23
33	clobetasol propionate (丙酸氯倍他索)			6.797			467.0		355.2^*^		13	17	17
									373.1		22	13	25
34	clobetasone butyrate (氯倍他松丁酸酯)			7.144			479.1		279.1^*^		13	21	18
									343.2		13	19	25
35	betamethasone 17-valerate (倍他米松戊酸酯)			6.971			477.1		279.2^*^		17	20	30
									355.2		17	14	17
36	betamethasone 17,21-dipropionate (倍他米松双丙酸酯)			7.175			505.3		279.1^*^		20	21	13
									319.1		24	19	11
37	prednicarbate (泼尼卡酯)			6.971			489.2		381.2^*^		27	13	19
									114.9		15	18	23
38	halcinonide (哈西奈德)			6.845			455.2		105.1^*^		14	52	11
									121.1		15	37	22
39	amcinonide (安西奈德)			7.066			503.2		321.1^*^		28	19	22
									339.2		28	17	24
40	alclometasone dipropionate (阿氯米松双丙酸酯)			6.657			521.1		319.2^*^		26	20	19
									301.2		28	21	10
41	beclomethasone dipropionate (倍氯米松双丙酸酯)			7.396			521.1		319.2^*^		24	13	26
									301.2		26	15	17
42	estradiol benzoate (苯甲酸雌二醇)			5.557			377.2		135.1^*^		17	25	28
									105.1		11	39	21
43	17*α*-hydroxyprogesterone (羟基孕酮)			6.024			331.2		109.1^*^		12	33	20
									97.1		14	28	10
44	medroxyprogesterone acetate (醋酸甲羟孕酮)			6.991			387.2		123.1^*^		18	14	24
									327.3		10	29	25
45	chlormadinone acetate (醋酸氯地孕酮)			5.494			405.1		309.3^*^		11	17	22
									301.2		15	21	30
46	megestrol acetate (醋酸甲地孕酮)			6.929			385.2		267.2^*^		14	20	19
									224.2		16	27	16
47	premarin (甲羟孕酮)			6.737			345.3		123.1^*^		10	27	22
									97.1		15	27	19
48	norethindrone (炔诺酮)			5.916			299.2		231.2^*^		15	21	29
									109.1		12	28	23
49	stanozolol (康力龙)			7.382			329.4		121.1^*^		13	39	25
									81.1		10	49	16
50	progesterone (孕酮)			7.052			315.1		97.1^*^		24	21	19
									109.1		11	27	22
51	nandrolone (诺龙)			5.932			275.2		257.2^*^		15	16	19
									239.2		12	17	25
52	nandrolone phenylpropionate (苯丙酸诺龙)			8.613			407.2		257.2^*^		16	16	18
									239.2		16	21	25
53	testosterone (睾酮)			6.213			289.2		109.1^*^		11	23	21
									97.2		11	23	21
54	boldenone (勃地酮)			6.731			287.2		121.1^*^		11	23	23
									135.2		11	14	14
55	trenbolone (群勃龙)			5.637			271.1		153.2^*^		14	49	16
									199.1		11	22	14
56	17-methyltestosterone (甲基睾酮)			6.55			303.1		109.1^*^		11	27	22
									97.1		11	28	10
57	androstenedione (雄烯二酮)			7.482			287.2		109.1^*^		20	24	22
									97.1		12	22	21
58	metandienone (美雄酮)			6.042			301.2		149.2^*^		12	16	15
									121.1		13	24	23
59	testosterone propionate (丙酸睾酮)			8.044			345.2		109.1^*^		13	27	23
									97.1		14	27	20
60	levonorgestrel (左炔诺孕酮)			6.448			313.2		109.1^*^		16	26	11
									245.2		12	18	17

* Quantitative ion.

### 1.2 样品前处理

分别准确称取粉碎后的固体或半固体样品2 g,液体样品(运动营养液)5 g,加入5 mL水(液体样品除外),涡旋混匀后再加入10 mL 1.0%(v/v)甲酸乙腈,于超声波清洗机中提取20 min,加入3 g氯化钠粉末摇匀,再以4500 r/min离心3 min,准确移取上层清液6.0 mL于塑料离心管中,加入PSA和C_18_粉末各150 mg,涡旋净化30 s后以4500 r/min离心3 min,再准确移取5.0 mL净化液于玻璃刻度管中,在40 ℃水浴中氮吹浓缩至近干,加入1.00 mL 50%甲醇水溶液溶解残渣,涡旋混匀过0.22 μm微孔有机滤膜后经UPLC-MS/MS测定。

### 1.3 标准溶液的配制

分别准确吸取质量浓度均为1000 μg/mL的60种类固醇激素标准溶液,用甲醇稀释成质量浓度均为2.0 μg/mL的混合标准工作液。再准确吸取一定量的混合标准工作液,用50%甲醇水稀释成质量浓度为1.00~100 ng/mL的系列混合标准工作溶液。

### 1.4 色谱-质谱条件

#### 1.4.1 色谱条件

色谱柱为Waters Acquity UPLC HSS T_3_ (100 mm×2.1 mm, 1.8 μm);流动相A为0.1%(v/v)甲酸水溶液(含10 mmol/L乙酸铵),流动相B为甲醇;柱温为40 ℃;流速为0.3 mL/min;进样量为5 μL;梯度洗脱程序:0~7.00 min, 40%B~95%B; 7.00~9.00 min, 95%B; 9.00~9.01 min, 95%B~40%B; 9.01~11.00 min, 40%B。

#### 1.4.2 质谱条件

电喷雾离子源(ESI),正离子、多反应监测模式(MRM);接口电压为4000 V;加热块温度为400 ℃;雾化气流速为3 L/min;干燥气流速为10 L/min。60种化合物的定性、定量离子对及其他质谱参数见表1。

## 2 结果与讨论

### 2.1 分析条件的优化

#### 2.1.1 质谱条件的优化

在ESI源、正离子扫描模式下,采用流动注射模式(FIA)将质量浓度均为2.0 μg/mL的混合标准工作溶液进行母离子扫描,确定60种目标物的母离子峰均为[M+H]^+^;再进行产物离子扫描,获得各化合物的碎片离子,根据欧盟2002/657/EC决议质谱分析方法中不得小于4个识别点的规定,并结合文献[12,27,31]选择响应值较高的2个碎片离子分别作为定量、定性离子;最后采用多反应监测模式进行参数优化,优化后的质谱参数见表1。

#### 2.1.2 色谱条件的优化

类固醇激素是一类含有环戊烷多氢菲母核的化合物,60种化合物中含有4对同分异构体,具有相同的母离子和碎片离子,质谱无法将其区分,需要优化色谱条件将同分异构体完全分离,才能进行准确定性、定量。文献[27⇓-29,31]采用C_18_填料的色谱柱进行分析,本实验对比了两种效果较好的Waters Acquity UPLC HSS T_3_ (100 mm×2.1 mm, 1.8 μm)和Waters Acquity UPLC BEH C_18_ (100 mm×2.1 mm, 1.7 μm)色谱柱。结果显示,两种色谱柱对目标物均具有较好的保留,但相比于BEH C_18_柱,HSS T_3_色谱柱获得的峰形更尖锐对称,响应也更高,确定为本方法的色谱柱。

对于电喷雾正离子模式,流动相中加入甲酸可以提高目标物的质子化效率,从而提高灵敏度。陈晓鹏等^[12]^在分析类固醇激素时,对流动相中甲酸的体积分数(0.05%~0.3%)进行了优化,发现在0.05%~0.1%时,目标物的响应值逐渐增加;而超过0.1%时,响应值没有明显变化而基线明显升高。在流动相中加入甲酸铵、乙酸铵等缓冲盐可以提高目标物的分离度以及峰形。本实验对比了甲醇-0.1%甲酸水溶液、乙腈-0.1%甲酸水溶液、甲醇-0.1%甲酸水溶液(含10 mmol/L乙酸铵)、乙腈-0.1%甲酸水溶液(含10 mmol/L乙酸铵)4种流动相。结果显示,当采用甲醇-0.1%甲酸水溶液(含10 mmol/L乙酸铵)作为流动相时,60种类固醇激素具有更好的响应和峰形,且通过优化梯度洗脱程序,能够实现4对同分异构体的基线分离,60种化合物的总离子流色谱图见图1, 4对同分异构体的提取离子色谱图见图2。

**图 1:**
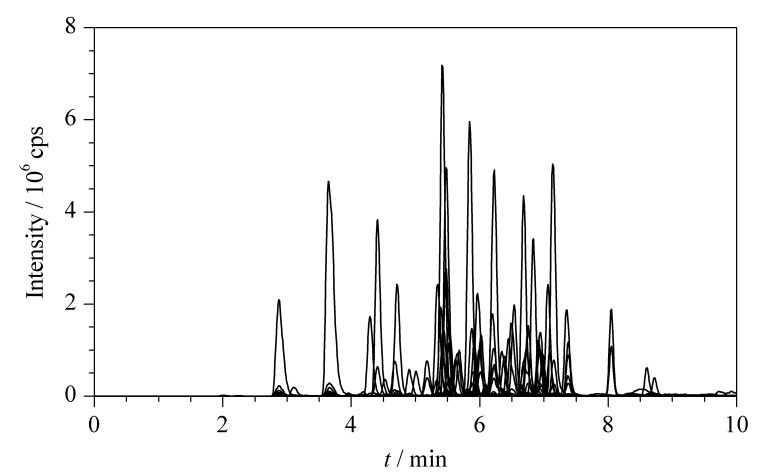
60种类固醇激素的总离子流色谱图

**图 2:**
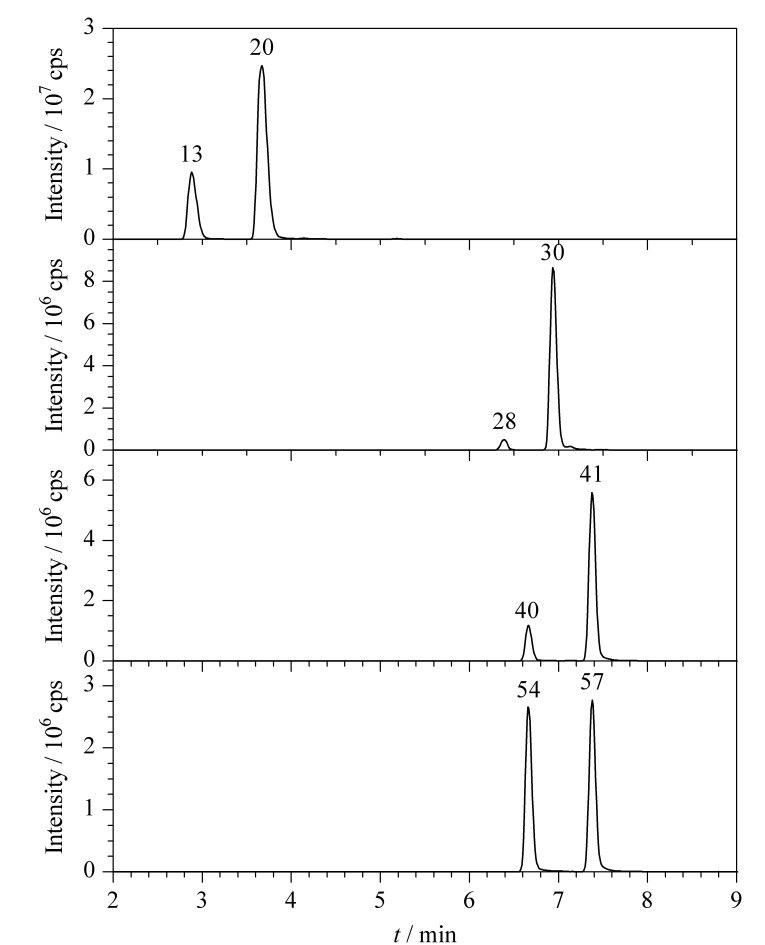
4对同分异构体的提取离子色谱图

### 2.2 样品前处理条件的优化

#### 2.2.1 提取溶剂的选择

类固醇激素极性较弱,易溶于有机溶剂中,陈晓鹏等^[12]^和赵倩茹等^[31]^等对比了丙酮、乙酸乙酯、甲醇、乙腈作为提取溶剂的效果,均认为乙腈最佳。考虑到直接采用乙腈提取无法完全渗透样品基质,本实验先加入水将样品基质分散后,再对比乙腈、1%(v/v)甲酸乙腈、1%(v/v)氨水乙腈3种提取溶剂对60种化合物回收率的影响。结果见图3,乙腈和1%(v/v)氨水乙腈效果相当,回收率分别为60.8%~113.4%、60.2%~112.9%,平均回收率分别为73.4%和74.7%;而1%(v/v)甲酸乙腈提取效果较好,回收率为61.7%~118.0%,平均值为85.0%。这是因为大多数类固醇化合物属于酯类,酸性的提取溶剂会抑制其水解,提取效率更高。因此选择1%(v/v)甲酸乙腈作为提取溶剂。

**图 3:**
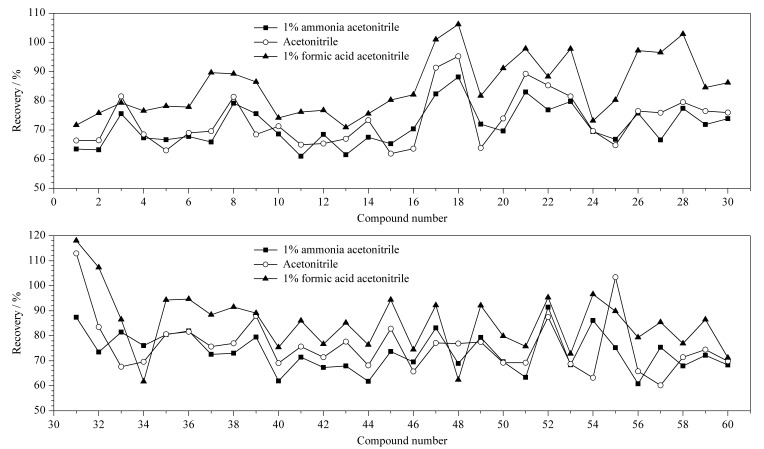
提取溶剂对类固醇激素回收率的影响

#### 2.2.2 QuEChERS净化填料的选择

运动营养食品中含有油脂、蛋白质、磷脂等杂质,会对色谱柱、质谱造成污染并产生基质效应,影响定量结果的准确性,需进行净化处理。QuEChERS方法中常用的净化剂有C_18_、PSA和GCB。C_18_吸附剂主要用于除去一些非极性杂质及油脂类物质;PSA吸附剂能有效去除糖类、脂肪酸等极性干扰物;而GCB因对含有苯环结构的化合物及弱极性化合物均有较强的吸附作用,容易造成该结构类型分析物回收率的降低,因此不适合本文所涉及的大多数目标物的净化。C_18_、PSA两种净化剂用量太少达不到净化要求,而用量太多则有可能会吸附目标物,因此,必须对吸附剂用量进行优化。实验采用10.0 ng/mL的混合标准溶液,分别单独考察了不同用量(50、100、150、200、250、300 mg)的PSA、C_18_作为吸附剂时的净化效果。结果显示,当PSA用量超过150 mg时,泼尼松、泼尼松龙、可的松、氢化可的松4种化合物有不同程度的吸附,回收率降为55.8%~87.8%(见图4a),因此选择PSA用量为150 mg; C_18_吸附剂超过150 mg时,地塞米松醋酸酯、地夫可特、孕酮、氢化可的松戊酸酯、倍他米松戊酸酯、醋酸甲地孕酮6种化合物有吸附,回收率降为54.8%~87.9%(见图4b),因此选择C_18_用量为150 mg。

**图 4:**
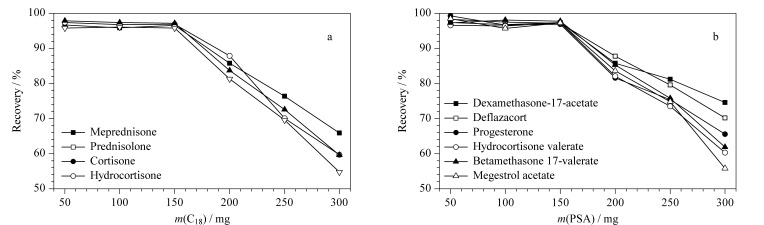
QuEChERS净化填料用量对类固醇激素回收率的影响

#### 2.2.3 净化方式的对比

文献报道的类固醇激素的净化方式主要有固相萃取法^[11,19,20,25]^和QuEChERS^[12,18,29]^净化技术。适用于类固醇激素净化的固相萃取柱可分为两种,一种是传统的保留目标物的原理,如HLB柱,另一种则是保留杂质的原理,如PRIME HLB柱。为了探索最佳的净化方式,本实验选用乳清蛋白粉为基质,在添加水平为10 μg/kg的条件下考察3种净化方式的具体效果。结果如图5所示,两种固相萃取柱的萃取效果接近,对于泼尼松龙、地塞米松、曲安奈德、布地奈德、倍他米松双丙酸酯、羟基孕酮、睾酮、丙酸睾酮的回收率较差,均低于60%;而采用QuEChERS净化时,60种化合物的回收率为73.7%~114.7%,满足分析要求。此外,采用固相萃取柱净化时,相对标准偏差也更大,重复性较差。因此选择QuEChERS作为净化方法。

**图 5:**
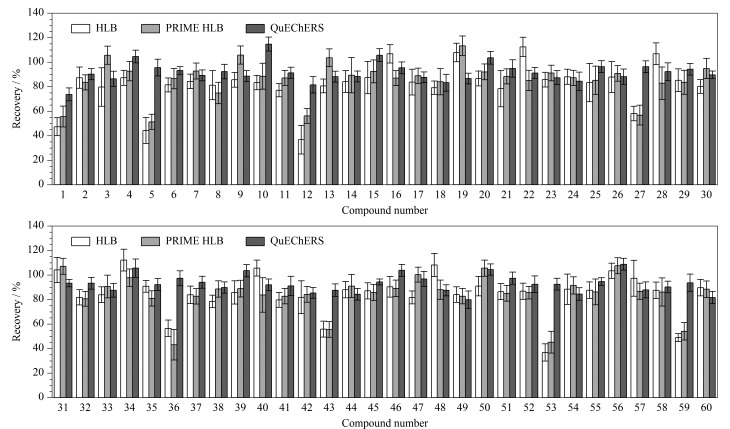
净化方式对类固醇激素回收率的影响(*n*=3)

#### 2.2.4 定容液的选择

净化后的溶液为1%甲酸乙腈,与初始流动相(40%甲醇水溶液)差距较大,会产生溶剂效应影响色谱峰峰形^[31]^,必须转换溶剂。以乳清蛋白粉为基质进行10 μg/kg的加标回收试验,考察不同体积分数甲醇水溶液作为定容液的效果。结果发现,当定容液中甲醇体积分数低于50%时,一些化合物回收率偏低,尤其是保留较强的化合物(见图6),

**图 6:**
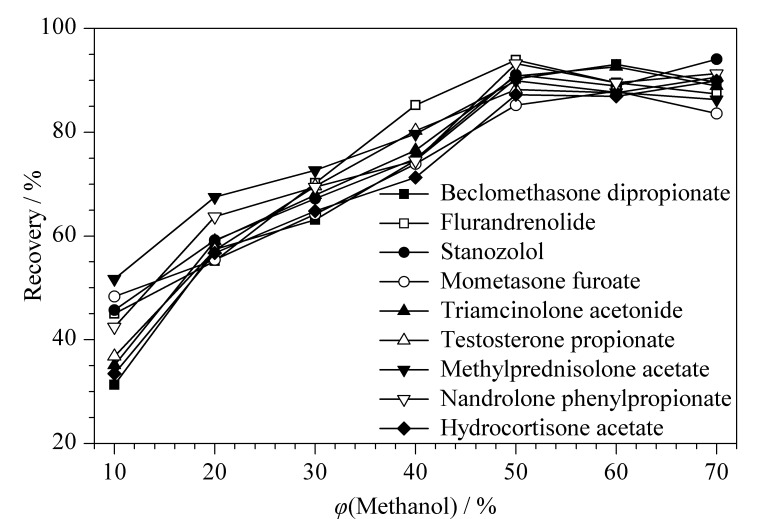
含不同体积分数甲醇的水溶液作为定容液对类固醇激素回收率的影响

这是由于类固醇激素化合物的极性较弱,易溶解在有机溶剂中,低比例的甲醇无法将其完全溶解;而从峰形考虑,当甲醇体积分数高于50%时,保留较弱的化合物出现前伸峰以及峰形变宽的典型溶剂效应现象,因此选择50%甲醇水作为定容液。

### 2.3 基质效应评价

采用电喷雾离子源质谱分析时,目标化合物的离子化效率会受到样品种类、前处理条件、仪器条件的影响,须对基质效应(matrix effect, ME)进行评价。分别选取乳清蛋白粉、果胶蛋白肽、运动营养液样品进行前处理得到空白样液,按照ME=(基质匹配标准溶液峰面积/溶剂标准溶液峰面积-1)×100%计算,ME在±20%以内表示基质效应不明显^[32]^。结果如图7所示,60种类固醇激素的ME为3.1%~13.5%,说明该前处理方法下基质效应较低,符合分析要求。

**图 7:**
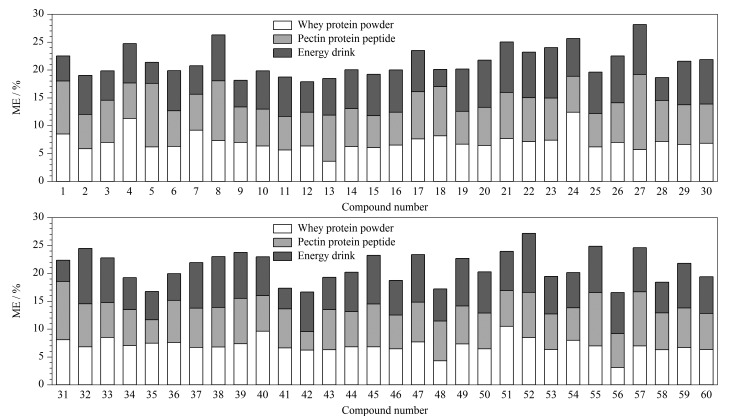
类固醇激素的基质效应

### 2.4 方法学考察

#### 2.4.1 线性范围、检出限和定量限

在优化的条件下,将系列混合标准工作溶液上机测试,以各自化合物定量离子的峰面积为纵坐标(*Y*),对应的质量浓度为横坐标(*X*, ng/mL)绘制标准曲线。结果显示,60种类固醇激素在1.00~100 ng/mL范围内线性关系良好,相关系数(*r*)均高于0.99。分别称取空白乳清蛋白粉2 g、空白果胶蛋白肽2 g、运动营养液5 g,进行加标回收试验,以*S/N*≥3和*S/N*≥10确定各化合物在对应样品基质中方法的检出限(LOD)和定量限(LOQ),分别为0.2~0.8 μg/kg和0.6~2.4 μg/kg,具体结果见表2。

**表2:** 60种类固醇激素的线性方程、相关系数、检出限和定量限

No.		Linear equation		*r*		LODs/(μg/kg)								LOQs/(μg/kg)				
						Whey protein powder		Pectin protein peptide		Energy drink				Whey protein powder		Pectin protein peptide		Energy drink
1		*y*=2.69×10^4^*x*+3.40×10^4^		0.997		0.6		0.6		0.3				1.8		1.9		0.9
2		*y*=1.10×10^5^*x*+8.86×10^4^		0.993		0.8		0.6		0.4				2.4		1.8		1.2
3		*y*=2.81×10^4^*x*+5.80×10^4^		0.990		0.5		0.5		0.5				1.5		1.6		1.4
4		*y*=1.07×10^4^*x*+9.31×10^4^		0.997		0.6		0.6		0.5				1.8		1.9		1.5
5		*y*=8.98×10^3^*x*+1.39×10^4^		0.988		0.4		0.4		0.4				1.2		1.3		1.1
6		*y*=1.87×10^4^*x*+3.70×10^5^		0.993		0.6		0.6		0.6				1.8		1.9		1.7
7		*y*=1.17×10^4^*x*+1.08×10^4^		0.998		0.3		0.3		0.3				0.9		1.0		0.8
8		*y*=1.09×10^4^*x*+9.74×10^4^		0.995		0.4		0.4		0.4				1.2		1.3		1.1
9		*y*=1.66×10^4^*x*+6.96×10^4^		0.997		0.8		0.6		0.5				2.4		1.8		1.5
10		*y*=7.17×10^4^*x*+6.17×10^4^		0.994		0.2		0.2		0.2				0.6		0.6		0.6
11		*y*=7.61×10^4^*x*+4.71×10^4^		0.991		0.4		0.4		0.4				1.2		1.3		1.1
12		*y*=5.01×10^4^*x*+2.24×10^4^		0.991		0.4		0.4		0.4				1.2		1.3		1.1
13		*y*=1.77×10^4^*x*+2.27×10^4^		0.997		0.7		0.5		0.4				2.1		1.5		1.3
14		*y*=7.82×10^4^*x*+5.42×10^4^		0.999		0.5		0.5		0.5				1.5		1.6		1.4
15		*y*=2.86×10^4^*x*+3.18×10^4^		0.996		0.3		0.3		0.3				0.9		1.0		0.8
16		*y*=7.67×10^5^*x*+3.41×10^5^		0.999		0.4		0.4		0.4				1.2		1.3		1.1
17		*y*=6.26×10^5^*x*+8.36×10^4^		0.995		0.5		0.5		0.5				1.5		1.6		1.4
18		*y*=3.81×10^4^*x*+6.41×10^4^		0.996		0.4		0.4		0.4				1.2		1.3		1.1
19		*y*=9.29×10^4^*x*+1.09×10^5^		0.993		0.5		0.5		0.5				1.5		1.6		1.4
20		*y*=1.72×10^5^*x*+3.52×10^4^		0.999		0.5		0.5		0.5				1.5		1.6		1.4
21		*y*=2.34×10^4^*x*+1.33×10^4^		0.998		0.7		0.6		0.5				2.1		1.8		1.5
22		*y*=1.34×10^5^*x*+1.38×10^4^		0.995		0.6		0.6		0.6				1.8		1.9		1.7
23		*y*=6.42×10^4^*x*+6.68×10^4^		0.998		0.4		0.4		0.4				1.2		1.3		1.1
24		*y*=1.98×10^4^*x*+1.74×10^4^		0.996		0.6		0.5		0.4				1.8		1.5		1.3
25		*y*=8.79×10^4^*x*+1.52×10^4^		0.997		0.6		0.6		0.5				1.8		1.9		1.5
26		*y*=8.46×10^4^*x*+4.06×10^4^		0.999		0.6		0.4		0.3				1.8		1.2		1.0
27		*y*=3.22×10^4^*x*+1.09×10^4^		0.998		0.6		0.6		0.4				1.8		1.9		1.2
28		*y*=4.51×10^4^*x*+4.85×10^4^		0.993		0.5		0.5		0.5				1.5		1.6		1.4
29		*y*=1.01×10^4^*x*+3.20×10^4^		0.999		0.4		0.4		0.4				1.2		1.3		1.1
30		*y*=4.36×10^4^*x*+1.91×10^4^		0.993		0.6		0.6		0.6				1.8		1.9		1.7
31		*y*=1.99×10^4^*x*+2.80×10^4^		0.998		0.7		0.5		0.4				2.1		1.5		1.2
32		*y*=1.65×10^4^*x*+2.71×10^4^		0.996		0.5		0.5		0.5				1.5		1.6		1.4
33		*y*=7.89×10^4^*x*+1.32×10^5^		0.996		0.4		0.4		0.4				1.2		1.3		1.1
34		*y*=7.87×10^4^*x*+1.40×10^4^		0.999		0.5		0.5		0.5				1.5		1.6		1.4
35		*y*=2.00×10^4^*x*+7.61×10^4^		0.999		0.6		0.6		0.6				1.8		1.9		1.7
36		*y*=1.07×10^4^*x*+7.99×10^4^		0.994		0.7		0.5		0.4				2.1		1.5		1.3
37		*y*=2.36×10^4^*x*+1.19×10^4^		0.999		0.3		0.3		0.3				0.9		1.0		0.8
38		*y*=2.38×10^4^*x*+1.90×10^4^		0.997		0.7		0.6		0.5				2.1		1.8		1.5	
39		*y*=1.01×10^5^*x*+6.94×10^4^		0.995		0.5		0.5		0.5				1.5		1.6		1.4
40		*y*=7.42×10^4^*x*+6.78×10^4^		0.998		0.6		0.6		0.4				1.8		1.9		1.2
41		*y*=2.06×10^5^*x*+7.55×10^4^		0.999		0.6		0.6		0.6				1.8		1.9		1.7
42		*y*=1.01×10^5^*x*+1.89×10^4^		0.997		0.7		0.5		0.5				2.1		1.5		1.5
43		*y*=1.51×10^4^*x*+1.39×10^5^		0.993		0.4		0.4		0.4				1.2		1.3		1.1
44		*y*=1.59×10^5^*x*+3.72×10^4^		0.999		0.5		0.5		0.5				1.5		1.6		1.4
45		*y*=5.17×10^4^*x*+7.31×10^4^		0.992		0.6		0.6		0.6				1.8		1.9		1.7
46		*y*=4.25×10^4^*x*+1.51×10^4^		0.998		0.6		0.6		0.6				1.8		1.9		1.7
47		*y*=4.80×10^5^*x*+2.48×10^4^		0.999		0.4		0.4		0.4				1.2		1.3		1.1
48		*y*=8.54×10^4^*x*+4.71×10^4^		0.999		0.6		0.4		0.3				1.8		1.2		1.0
49		*y*=5.93×10^4^*x*-1.17×10^4^		0.999		0.5		0.5		0.5				1.5		1.6		1.4	
50		*y*=5.96×10^5^*x*-2.68×10^4^		0.999		0.7		0.6		0.5				2.1		1.8		1.5
51		*y*=7.87×10^4^*x*-4.95×10^4^		0.992		0.4		0.4		0.4				1.2		1.3		1.1
52		*y*=2.78×10^4^*x*-1.59×10^4^		0.996		0.6		0.6		0.6				1.8		1.9		1.7
53		*y*=2.74×10^4^*x*+2.26×10^4^		0.998		0.5		0.5		0.5				1.5		1.6		1.4
54		*y*=3.74×10^4^*x*+6.34×10^4^		0.996		0.6		0.6		0.4				1.8		1.9		1.2
55		*y*=7.24×10^4^*x*+1.00×10^5^		0.991		0.6		0.6		0.6				1.8		1.9		1.7
56		*y*=3.40×10^4^*x*+1.32×10^5^		0.996		0.8		0.6		0.3				2.4		1.8		0.9
57		*y*=3.38×10^5^*x*-3.30×10^4^		0.999		0.5		0.5		0.5				1.5		1.6		1.4
58		*y*=1.56×10^4^*x*+1.93×10^4^		0.998		0.5		0.5		0.5				1.5		1.6		1.4
59		*y*=3.27×10^5^*x*-2.51×10^4^		0.999		0.7		0.5		0.4				2.1		1.5		1.3
60		*y*=2.24×10^4^*x*+6.98×10^4^		0.999		0.7		0.6		0.5				2.1		1.8		1.5

For compound Nos., see Table 1. *y*: peak area; *x*: mass concentration, ng/mL.

#### 2.4.2 回收率和精密度

分别对空白乳清蛋白粉、果胶蛋白肽、运动营养液进行低、中、高3个水平的加标回收试验(*n*=6)。结果见表3, 60种化合物的平均回收率为73.7%~112.7%, RSD为3.2%~10.1%,说明该方法的准确度和重复性较好。

**表3:** 3种基质中60种类固醇激素的加标回收率与RSD(*n*=6)

No.	Added/(μg/kg)			Whey protein powder						Pectin protein peptide					Energy drink		
				Recovery/%		RSD/%				Recovery/%		RSD/%			Recovery/%	RSD/%	
1		2.50	76.5		6.0				76.8		4.6				78.9		5.8
		5.00	81.3		4.1				86.0		6.6				87.9		4.5
		20.0	84.8		4.8				81.2		6.5				88.7		6.4
2		2.50	91.6		4.5				90.3		5.2				85.1		4.4
		5.00	89.5		4.5				85.6		7.8				93.1		5.1
		20.0	90.4		4.8				90.6		4.8				90.9		4.8
3		2.50	81.5		6.0				82.5		5.2				86.9		4.8
		5.00	85.1		6.2				85.8		6.6				93.5		5.1
		20.0	89.3		4.6				87.9		6.9				86.4		6.4
4		2.50	103.6		7.1				108.2		6.0				102.3		6.8
		5.00	107.8		6.8				97.5		7.7				93.5		4.9
		20.0	101.3		5.6				110.3		7.4				107.8		7.6
5		2.50	84.7		4.2				83.5		6.1				86.0		7.3
		5.00	88.4		5.4				87.1		4.6				86.0		6.0
		20.0	91.4		6.8				90.1		5.8				89.8		4.5
6		2.50	84.1		6.6				84.8		7.4				93.0		5.7
		5.00	87.9		5.5				86.6		7.1				85.4		7.3
		20.0	83.1		6.8				81.9		5.9				89.4		7.0
7		2.50	86.2		7.1				87.5		7.4				84.4		5.8
		5.00	91.1		5.0				89.7		7.7				87.7		7.3
		20.0	87.5		3.2				86.1		5.4				92.5		7.6
8		2.50	83.8		4.2				82.8		3.5				88.9		5.4
		5.00	90.1		5.6				88.8		4.6				85.1		3.5
		20.0	92.5		5.7				85.0		6.1				91.6		4.5
9		2.50	87.6		4.8				86.2		6.2				93.9		6.0
		5.00	92.3		3.2				88.5		5.2				89.0		6.1
		20.0	88.5		7.8				87.2		3.5				93.8		5.1
10		2.50	109.3		6.2				91.4		8.5				103.3		3.5
		5.00	103.5		6.9				107.4		6.9				94.5		8.4
		20.0	106.3		5.4				102.8		7.5				105.7		6.8
11		2.50	86.0		4.8				84.7		5.8				94.4		7.4
		5.00	84.1		6.0				92.4		5.2				87.4		5.7
		20.0	89.5		7.5				88.2		6.6				85.4		5.1
12		2.50	84.1		6.9				82.8		8.1				91.0		6.4
		5.00	86.2		7.1				93.1		7.5				85.4		8.0
		20.0	88.5		6.8				87.2		7.7				87.7		7.4
13		2.50	93.3		5.7				83.1		7.4				89.9		7.6
		5.00	90.3		6.4				89.0		6.2				94.8		7.3
		20.0	86.1		5.1				84.9		7.0				91.7		6.1
14		2.50	92.4		7.1				91.1		5.6				87.6		6.9
		5.00	88.2		4.5				91.3		7.7				93.9		5.5
		20.0	87.4		5.4				86.0		4.8				89.6		7.6
15		2.50	101.2		5.9				99.5		5.8				103.5		4.8
		5.00	93.5		7.9				105.6		6.4				95.3		5.7
		20.0	106.8		6.1				92.1		8.6				104.8		6.3
16		2.50	90.4		6.8				93.5		6.7				88.7		8.5
		5.00	89.5		5.0				88.2		7.4				91.9		6.6
		20.0	93.0		6.2				91.5		5.4				91.0		7.3
17		2.50	84.0		4.5				82.6		6.9				94.4		5.4
		5.00	85.7		7.6				90.4		4.8				85.3		6.8
		20.0	86.3		8.3				85.0		8.2				87.0		4.8
18		2.50	93.4		6.8				89.5		9.0				87.7		8.1
		5.00	88.8		7.5				87.5		7.4				94.9		8.8
		20.0	89.3		3.2				87.9		8.1				90.2		7.3
19		2.50	73.7		7.3				84.3		5.0				79.2		6.4
		5.00	90.2		4.9				86.4		7.1				88.4		4.9
		20.0	86.4		5.8				86.6		4.9				89.1		7.1
20		2.50	104.6		5.4				108.7		5.7				95.3		4.8
		5.00	95.5		5.4				100.3		5.2				92.4		5.6
		20.0	104.5		5.8				105.3		5.2				91.3		5.2
21		2.50	89.1		7.3				90.8		5.7				87.3		5.2
		5.00	92.3		7.5				84.2		7.1				90.4		5.6
		20.0	88.7		5.5				88.4		7.5				86.8		7.1
22		2.50	112.7		8.5				105.2		5.4				91.1		7.4
		5.00	106.4		8.2				100.4		8.4				89.4		5.4
		20.0	104.3		6.8				97.8		8.0				82.7		8.3
23		2.50	88.2		5.0				83.9		6.7				86.4		8.0
		5.00	88.3		6.5				87.5		5.0				86.5		6.6
		20.0	105.7		8.2				90.5		6.3				90.3		4.9
24		2.50	95.4		7.9				85.2		8.0				93.4		6.3
		5.00	87.6		6.7				87.0		7.7				85.8		8.0
		20.0	83.2		8.2				82.3		6.4				89.8		7.6
25		2.50	86.6		8.5				87.9		8.0				84.8		6.4
		5.00	103.6		6.0				90.2		8.4				88.1		8.0
		20.0	94.9		3.9				86.5		5.9				92.9		8.3
26		2.50	91.2		5.0				83.2		3.8				89.3		5.9
		5.00	87.3		6.8				89.2		5.0				104.5		3.9
		20.0	84.4		6.9				85.4		6.7				92.1		4.9
27		2.50	96.3		5.8				86.6		6.8				94.3		6.6
		5.00	88.2		3.9				88.9		5.7				89.4		6.7
		20.0	96.2		9.4				87.6		3.8				94.2		5.6
28		2.50	92.2		7.5				81.1		9.3				90.3		3.9
		5.00	94.2		8.3				88.6		7.5				105.4		6.2
		20.0	89.6		6.5				91.0		8.1				91.4		7.4
29		2.50	86.0		5.8				85.1		6.3				94.8		5.1
		5.00	86.8		7.3				92.8		5.7				87.8		6.3
		20.0	83.3		9.0				88.6		7.1				103.2		5.6
30		2.50	91.1		8.3				83.2		8.8				91.4		7.1
		5.00	88.9		8.5				93.6		8.1				85.8		8.8
		20.0	85.1		8.2				87.6		8.4				88.1		8.1
31		2.50	88.1		6.9				83.5		8.0				90.3		8.3
		5.00	84.6		7.8				89.4		6.8				95.3		8.0
		20.0	88.7		6.2				85.3		7.6				92.2		6.7
32		2.50	87.0		8.5				91.5		6.1				88.0		7.5
		5.00	80.5		5.4				91.8		8.4				94.3		6.0
		20.0	84.1		6.5				86.5		5.2				90.1		5.3
33		2.50	84.2		7.2				85.4		6.3				89.2		5.2
		5.00	87.9		9.5				86.9		7.0				83.6		6.3
		20.0	90.9		7.4				86.4		9.4				85.8		7.0
34		2.50	103.5		8.2				104.6		7.2				108.3		9.3
		5.00	94.5		6.0				93.9		8.0				93.4		7.2
		20.0	107.1		7.5				108.3		5.9				98.2		8.0
35		2.50	85.7		5.4				83.0		7.5				94.8		5.9
		5.00	90.5		9.2				90.8		5.2				85.7		7.4
		20.0	87.0		10.1				85.4		8.9				87.4		5.2
36		2.50	83.3		8.2				89.9		9.8				88.1		8.9
		5.00	89.6		9.0				87.9		8.0				95.4		4.7
		20.0	91.9		3.9				88.4		8.8				90.6		8.0
37		2.50	87.0		6.9				80.1		4.7				75.3		6.1
		5.00	91.7		4.7				82.0		6.8				83.9		4.7
		20.0	88.0		5.5				82.3		4.6				84.7		6.7
38		2.50	89.8		5.1				86.2		5.4				81.2		4.5
		5.00	89.0		5.1				81.7		4.9				88.9		5.3
		20.0	92.4		5.5				80.5		4.9				86.7		5.0
39		2.50	106.5		6.9				96.3		5.4				92.9		5.0
		5.00	98.4		7.2				100.0		6.8				95.9		5.3
		20.0	108.5		5.3				94.9		7.1				92.5		6.7
40		2.50	83.6		8.1				82.3		5.2				86.5		7.0
		5.00	85.7		7.8				81.0		7.9				84.9		5.1
		20.0	88.0		6.4				79.6		7.6				78.5		7.9
41		2.50	92.8		4.8				79.7		6.3				82.0		7.6
		5.00	89.8		6.2				83.1		4.7				82.1		6.3
		20.0	85.6		7.8				86.0		6.0				85.7		4.7
42		2.50	91.8		7.5				81.0		7.6				88.7		6.0
		5.00	87.7		6.3				82.7		7.3				81.5		7.6
		20.0	86.9		7.8				78.2		6.1				85.3		7.3
43		2.50	81.4		8.1				83.5		7.6				80.6		6.1
		5.00	83.6		5.7				85.6		7.9				83.7		7.6
		20.0	86.8		3.7				82.2		5.6				88.3		7.9
44		2.50	89.9		4.8				79.1		3.7				84.8		5.6
		5.00	89.0		6.4				84.7		4.7				81.2		3.7
		20.0	92.4		6.6				81.1		6.3				87.5		4.7
45		2.50	83.5		5.5				82.3		6.4				89.6		6.3
		5.00	85.2		3.7				84.5		5.4				84.9		6.4
		20.0	85.8		9.0				83.2		3.7				89.5		5.3
46		2.50	92.9		7.2				77.1		8.8				85.8		3.7
		5.00	88.3		7.9				84.2		7.1				87.6		8.8
		20.0	88.7		6.2				86.5		7.7				86.8		7.0
47		2.50	79.3		5.5				80.9		6.0				90.1		7.7
		5.00	84.8		6.9				88.2		5.4				83.4		6.0
		20.0	81.2		8.6				84.2		6.8				81.5		5.3
48		2.50	98.3		7.9				79.1		8.4				86.8		6.7
		5.00	89.8		8.1				88.9		7.7				81.5		8.3
		20.0	87.6		7.8				83.2		7.9				83.7		7.7
49		2.50	83.8		6.6				79.3		7.6				85.8		7.9
		5.00	86.8		7.4				84.9		6.4				90.5		7.6
		20.0	83.3		5.8				81.0		7.2				87.5		6.4
50		2.50	107.8		8.1				86.9		5.8				83.6		7.1
		5.00	85.8		5.1				87.2		7.9				89.6		5.7
		20.0	76.6		6.2				82.1		4.9				85.6		7.9
51		2.50	82.9		6.8				81.1		6.0				84.7		5.0
		5.00	83.0		9.1				82.6		6.7				79.4		6.0
		20.0	99.4		7.0				82.0		8.9				81.5		6.6
52		2.50	89.6		7.8				89.3		6.9				84.7		8.9
		5.00	82.3		5.7				84.2		7.6				87.7		6.8
		20.0	78.2		7.2				87.4		5.6				86.8		7.6
53		2.50	81.4		5.1				78.9		7.1				90.1		5.6
		5.00	97.4		8.7				86.3		4.9				81.4		7.0
		20.0	89.2		9.5				81.1		8.5				83.0		5.0
54		2.50	85.7		7.8				85.4		9.3				83.7		8.4
		5.00	82.1		8.6				83.5		7.6				90.6		9.2
		20.0	79.3		3.7				83.9		8.4				86.1		7.6
55		2.50	90.5		6.6				76.0		4.5				89.6		5.8
		5.00	82.9		4.4				77.9		6.4				93.5		4.4
		20.0	90.5		5.2				78.2		4.4				104.8		6.4
56		2.50	86.7		4.9				81.9		5.1				93.6		4.3
		5.00	88.5		4.9				77.6		4.7				102.5		5.0
		20.0	84.3		5.2				76.5		4.7				82.4		4.7
57		2.50	80.8		6.6				82.0		5.1				94.5		4.7
		5.00	81.6		6.8				76.0		6.4				91.3		5.0
		20.0	78.3		5.0				79.7		6.7				88.6		6.4
58		2.50	85.6		7.7				78.2		4.9				82.2		6.7
		5.00	83.6		7.4				77.0		7.6				80.7		4.8
		20.0	80.0		6.1				75.6		7.2				86.5		7.5
59		2.50	82.8		4.5				75.7		6.0				91.4		7.2
		5.00	79.5		5.9				79.0		4.5				85.9		6.0
		20.0	83.4		7.4				81.7		5.7				81.5		4.4
60		2.50	103.4		7.1				87.3		7.2				95.3		5.7
		5.00	89.7		6.0				96.4		6.9				89.4		7.2
		20.0	93.5		7.4				93.1		5.8				97.5		6.9

For compound Nos. 1-60, see Table 1.

此外,在同样条件下,将加标水平为2.50 μg/kg的样液每隔2 h进样一次,连续进样6次,以6次的回收率相对标准偏差计算日内精密度;同时每隔24 h进样一次,同样连续进样6次,以6 d所得回收率的相对标准偏差计算日间精密度。结果表明,60种类固醇化合物的日内精密度和日间精密度均较好,分别为1.5%~2.3%和2.4%~3.8%,说明类固醇类化合物在6 d内具有较好的稳定性,各方法学指标均符合实验室理化检测GB/T 27404-2008的方法学要求,适用于运动营养食品中类固醇激素的测定。

#### 2.4.3 与已报道方法的比较

与已报道的方法进行比较,结果见表4。文献报道的类固醇激素测定方法,主要针对的是燕窝、奶粉、牛奶、乳制品、牛蛙及化妆品,本研究针对的是运动营养食品(特殊膳食)。本方法较固相萃取法^[11,19,20,24]^消耗有机溶剂更少,仅需10 mL,且用时更少,检出限也较低,为0.2~0.8 μg/kg;文献[12,18]同样使用QuEChERS法,最多涉及42种类固醇激素。本方法涉及60种类固醇类激素,具有较高灵敏度的同时,前处理用时短、消耗有机溶剂少,是一种快速、环保的高通量分析方法。

**表4:** 本方法与已报道方法的比较

Sample	Number of compounds	Analytical method	Preparation method	Preparation time/min	*V*(Organic solvent)/mL	LOD/ (μg/kg)	Ref.
Bird’s nest	45	UPLC-MS/MS	SPE	>120	40	0.16-2.0	[11]
Milk powder	42	UPLC-MS/MS	QuEChERS	<60	10	0.06-1.5	[12]
Milk	9	UPLC-MS/MS	QuEChERS	<60	10	0.5-10	[18]
Dairy products	6	UPLC-MS/MS	SPE	>120	327	0.05-0.1	[19]
Bullfrogs	9	UPLC-MS/MS	SPE	>120	10	0.5-1.0	[20]
Cosmetics	22	UPLC-MS/MS	SPE	>120	20	0.1-27.4	[24]
Sports nutrition foods	60	UPLC-MS/MS	QuEChERS	<60	10	0.2-0.8	this work

### 2.5 实际样品测定

采用新开发的方法,对市售13批次运动营养食品进行60种类固醇激素的筛查,结果共检出5份阳性样品,其中4份乳清蛋白粉样品中检出内源性激素(孕酮),含量为12.6~25.4 μg/kg; 1份运动营养液中检出勃地酮,该药物属于运动员禁用药物,含量为14.6 μg/kg,说明运动营养食品中确有非法添加类固醇激素的现象,国家相关部门应加强对运动营养食品中该类物质的监控。

## 3 结论

本研究建立了超高效液相色谱-串联质谱法同时测定运动营养食品中60种类固醇激素的高通量方法。该方法采用QuEChERS的前处理方式,操作简单、高效,重复性好,灵敏度高,适用于运动营养食品中类固醇激素的快速高通量筛查以及定量分析,填补了当前运动营养食品中类固醇激素测定方法的空白,可为该类食品中类固醇激素的日常监督提供技术支撑。此外,利用本研究建立的方法对实际样品中类固醇类激素进行测定,结果表明运动营养食品中确有添加类固醇类激素的现象,希望国家相关部门尽快建立国家食品安全标准,对该类食品中类固醇激素进行日常监督。
